# Successful Surgical Management of Post-traumatic Superficial Femoral Artery Pseudoaneurysm With Delayed Presentation

**DOI:** 10.7759/cureus.45739

**Published:** 2023-09-21

**Authors:** Rajeshwar Yadav, Aditya Sharma, Swati Pathak

**Affiliations:** 1 Department of Cardiothoracic & Vascular Surgery, Institute of Medical Sciences, Banaras Hindu University, Varanasi, IND; 2 Department of General Surgery, Institute of Medical Sciences, Banaras Hindu University, Varanasi, IND

**Keywords:** vascular surgery, limb trauma, endovascular embolization, pseudoaneurysm, superficial femoral artery

## Abstract

Pseudoaneurysms of the superficial femoral artery are uncommon and typically iatrogenic. The majority of publications on this topic that have been published in English literature are case reports. It is unclear how often arterial pseudoaneurysms (APAs) caused by limb trauma occur, and their treatment is not standardised. A review of the literature usually follows the presentation of a case report, but no recent update with reliable data has been published.

In this case study, a 24-year-old patient who had a piece of iron lodged in the middle third of his thigh and a pulsatile mass that revealed a pseudoaneurysm of the superficial femoral artery is described. The patient underwent an emergency operation without employing a venous graft. We directly sutured the arterial lesion, with positive outcomes.

## Introduction

Following blunt trauma, pseudoaneurysms of the superficial femoral artery without any femoral fracture are a rare clinical phenomenon [1]. The majority of superficial femoral artery pseudoaneurysm cases present to the hospital after a penetrating injury, a femoral fracture, and surgical operations [2]. Depending on their size, some pseudoaneurysms might compress nearby structures, resulting in ischemia, haemorrhage, and even necrosis of the skin [3].

## Case presentation

We describe the case of a 24-year-old male patient who, one month prior to admission, met with an accident where a small metallic piece was lodged in the mid-thigh of his left lower limb, and it went unnoticed in the medical facility where he was treated earlier. This patient had no specific medical history, including no known comorbidities. A week later, the patient became aware of a progressively growing tumour that was associated with throbbing pain and consulted the superspeciality hospital for further workup. 

On inspection of the affected region, a pulsating swelling of 18x12 cm was noted in the left mid-thigh. On palpation, there was no local rise in temperature; the pulsating mass was soft in consistency and had irregular margins. No spontaneous bleeding occurred. Additionally, there was no evidence of limb ischemia, and all the peripheral pulses were palpable.

Doppler ultrasonography revealed a pseudoaneurysm of size 4.4x3.4x2.8 cm. The patient was planned for surgery on an elective basis, and immediately a contrast-enhanced computed tomography (CECT) of the left thigh along with magnetic resonance imaging (MRI) were performed. 

The CECT of the involved region suggested a well-defined hypodense collection with areas of layered blood attenuation seen in the mid-thigh intramuscular compartment involving the sartorius and adductor longus muscles, measuring 8.4x6.4x14.8 cm in size (mean CT attenuation value +60HU), and evidence of a large pseudoaneurysm arising from the mid-superficial femoral artery, with a neck measuring 4.4 mm and dome measuring 4.4x4.5 cm, with the distance of the pseudoaneurysm from the left hip joint being 20 cm.

**Figure 1:**
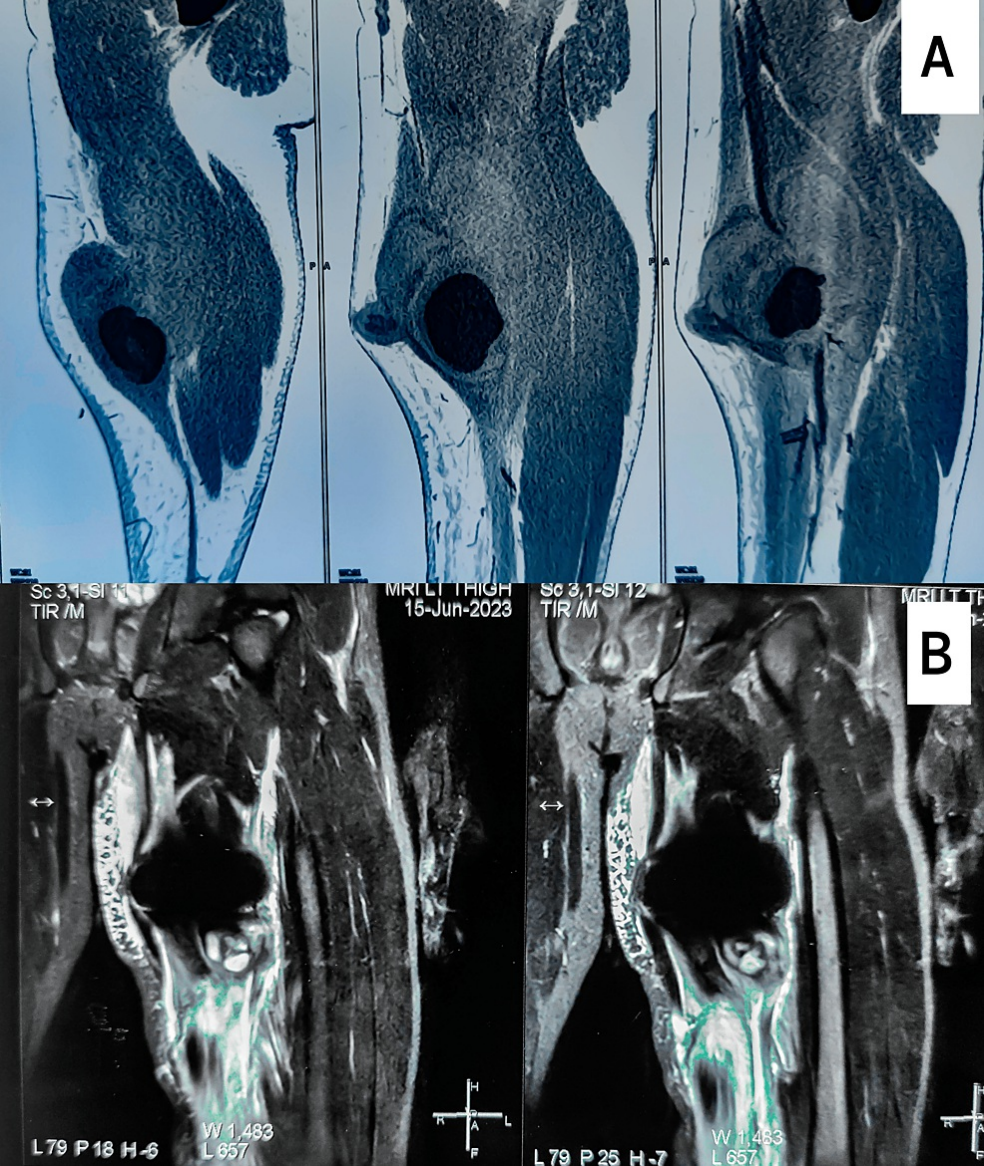
(A) CECT imaging of the left thigh showing well-defined hypodense collection with areas of layered blood attenuation seen in the mid-thigh intramuscular compartment involving the sartorius and adductor longus muscles, measuring 8.4x6.4x14.8 cm in size; (B) MRI of the left thigh showing significant intramuscular injury and hematoma formation along the tract of the foreign body in the adductor longus and adductor magnus muscles, with the foreign body lodged within the hamstring abutment, causing a significant metallic artefact. CECT: contrast-enhanced computed tomography, MRI: magnetic resonance imaging

The MRI was suggestive of significant intramuscular injury and hematoma formation along the tract of the foreign body in the adductor longus and adductor magnus muscles, with the foreign body lodged within the hamstring abutment, causing a significant metallic artefact, as shown in Figure 1A, 1B.

**Figure 2:**
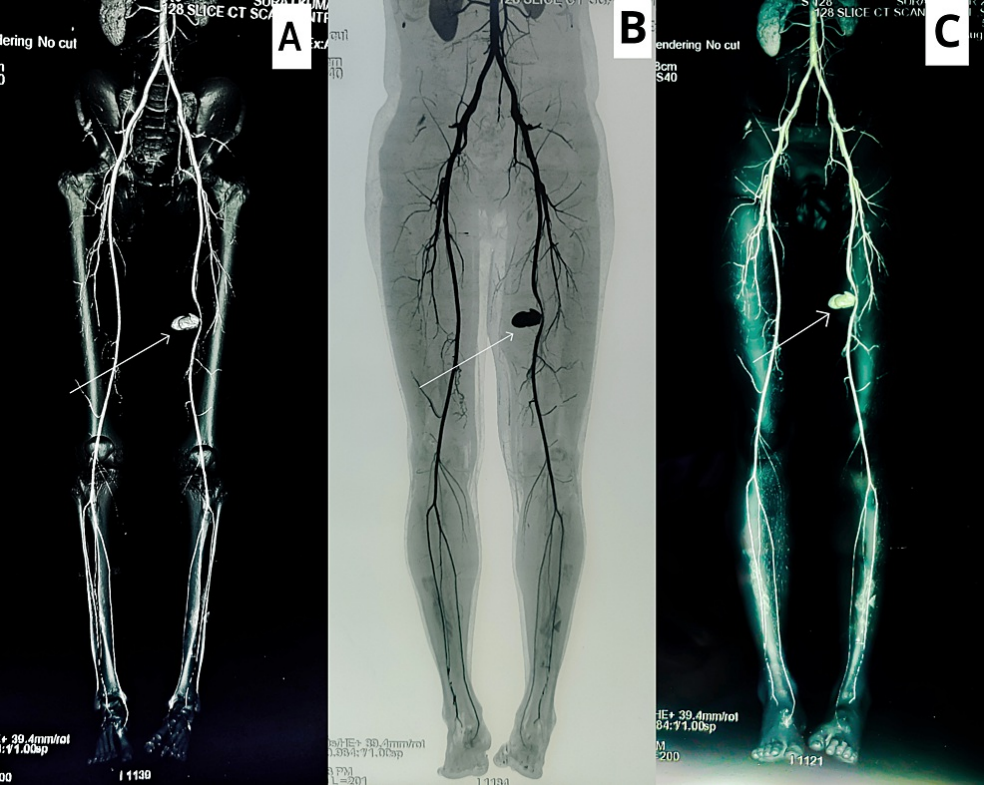
A pseudo-aneurysmal sac measuring 4.2x2.1x2.0 cm arising from the left superficial femoral artery in the mid-thigh. There was an associated large hematoma measuring 6.6x9.4x15.1 cm that communicated with the skin at the level of the mid-thigh. (A) the reconstruction of the arterial system in relation to the bones, (B) the reconstruction of arteries without bony and soft tissues and (C) the arterial system with bone and soft tissue.

On CT bilateral lower limb angiography, a pseudo-aneurysmal sac measuring 4.2x2.1x2.0 cm was noted arising from the left superficial femoral artery in the mid-thigh. There was an associated large hematoma measuring 6.6x9.4x15.1 cm that communicated with the skin at the level of the mid-thigh, as shown in Figure 2.

The patient underwent surgery because of the magnitude of the mass and the impending risk of rupture. We surgically accessed the mass from both sides and successfully managed an organised hematoma with several thrombi around the hematoma site. Intraoperatively, the patient was administered inj. heparin 5000 IU intravenous (IV) stat along with inj. sodabicarb 100 ml.

**Figure 3:**
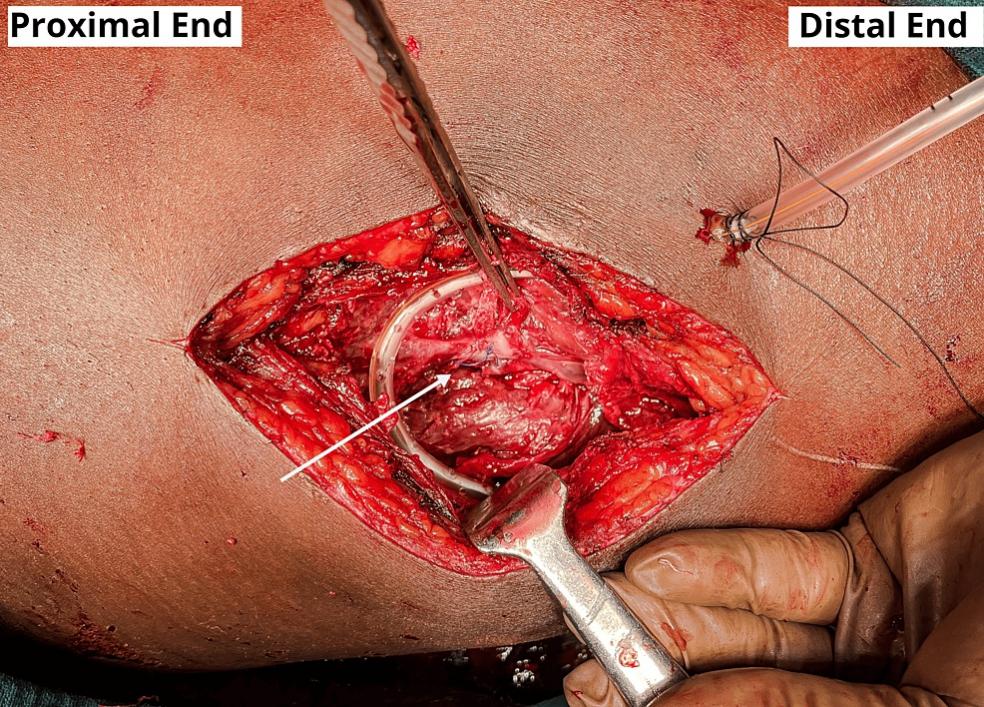
An intraoperative picture showing the arterial defect repaired directly without the use of any venous graft because the neck of the swelling was small and the arterial wall could be easily approximated and drain placement was done.

A 0.5 cm lesion was seen when the superficial femoral artery wall was examined. We repaired the arterial defect directly with prolene 5-0 sutures without using any venous graft because the neck of the swelling was small, the margins of the arterial wall were healthy and the arterial wall could be easily approximated and sutured, as shown in Figure 3.

The postoperative period was uneventful, and the patient was administered IV antibiotics, anti-inflammatory drugs, inj. microspan and inj. heparin 5000 IU at 40 mic drop per minute, low-dose antiplatelet drugs at the dose of 75 mg, tab warfarin 2 mg once daily, and phosphodiesterase type 3 inhibitors at a dose of 50 mg twice daily. On day five, the patient was discharged from the hospital, and the patient did well during his follow-up visits.

## Discussion

Doppler ultrasonography, which can assess the size, anatomy, and origin of pseudoaneurysms, continues to be the screening modality for diagnosis of such lesions [3]. If all the lesions cannot be accurately described, a CT scan may occasionally be recommended [4]. The biggest problem to worry about is pseudoaneurysm rupture, especially if size matters. A pseudoaneurysm manifests as the sensation of a pulsatile mass or thrill but may go unnoticed for a long time.

Continuous Doppler monitoring enables the suggestion of a cautious course of action [5]. Femoral artery pseudoaneurysms greater than 2.5 cm or exhibiting symptoms should be treated to avoid complications that could result in limb loss, such as rupture, thrombosis, or embolization [6]. Pseudoaneurysms can be managed with endovascular therapy or surgical correction. The possibility of thrombin injection in situ was mentioned in certain publications [7]. It has also been described as possible to treat these fictitious aneurysms with a covered stent, provided the anatomical parameters are suitable.

We specifically discuss this case because of the trauma's unanticipated mechanisms, following which a pseudoaneurysm developed. Although there have been a few reports of post-traumatic femoral artery pseudoaneurysms, the mechanism detailed in our patient's case is unique, as are the resulting anatomical abnormalities [8]. Since the pseudoaneurysm in our case measured 5.5 cm, we chose surgical management for immediate results and to prevent complications.

## Conclusions

Traumatic arterial pseudoaneurysms associated with limb trauma have become more prevalent over the past 10 years, and they are frequently identified following a painful presentation of swelling and/or pulsatile mass. The best choice for confirming the diagnosis is angiography. For the treatment of pseudoaneurysms originating from tiny branches of an artery, endovascular coiling is preferred. The standard of care for pseudoaneurysms originating from critical axial vessels is open surgical repair.
